# The Role of MicroRNAs in the Pathogenesis of Doxorubicin-Induced Vascular Remodeling

**DOI:** 10.3390/ijms252413335

**Published:** 2024-12-12

**Authors:** Ekaterina Podyacheva, Julia Snezhkova, Anatoliya Onopchenko, Vyacheslav Dyachuk, Yana Toropova

**Affiliations:** Almazov National Medical Research Centre, Ministry of Health of the Russian Federation, 197341 Saint-Petersburg, Russia or ekaterinapodyachevaspb@gmail.com (E.P.); snezhkova_yuv@almazovcentre.ru (J.S.); onopchenko_av@almazovcentre.ru (A.O.); dyachuk_va@almazovcentre.ru (V.D.)

**Keywords:** biomarker, doxorubicin, endothelium, heart disease, microRNA, tumor angiogenesis, vascular remodeling

## Abstract

Doxorubicin (DOX), a cornerstone chemotherapeutic agent, effectively combats various malignancies but is marred by significant cardiovascular toxicity, including endothelial damage, chronic heart failure, and vascular remodeling. These adverse effects, mediated by oxidative stress, mitochondrial dysfunction, inflammatory pathways, and dysregulated autophagy, underscore the need for precise therapeutic strategies. Emerging research highlights the critical role of microRNAs (miRNAs) in DOX-induced vascular remodeling and cardiotoxicity. miRNAs, such as miR-21, miR-22, miR-25, miR-126, miR-140-5p, miR-330-5p, miR-146, miR-143, miR-375, miR-125b, miR-451, miR-34a-5p, and miR-9, influence signaling pathways like TGF-β/Smad, AMPKa/SIRT, NF-κB, mTOR, VEGF, and PI3K/AKT/Nrf2, impacting vascular homeostasis, angiogenesis, and endothelial-to-mesenchymal transition. Despite existing studies, gaps remain in understanding the full spectrum of miRNAs involved and their downstream effects on vascular remodeling. This review synthesizes the current knowledge on miRNA dysregulation during DOX exposure, focusing on their dual roles in cardiovascular pathology and tumor progression. Strategies to reduce DOX cardiotoxicity include modulating miRNA expression to restore signaling balance, targeting pro-inflammatory and pro-fibrotic pathways, and leveraging miRNA inhibitors or mimics. This review aims to organize and integrate the existing knowledge on the role of miRNAs in vascular remodeling, particularly in the contexts of DOX treatment and the progression of various cardiovascular diseases, including their potential involvement in tumor growth.

## 1. Introduction

Doxorubicin (DOX) belongs to the anthracycline family of antibiotics. Approved for medical use since 1974, this chemotherapeutic agent is also listed among the World Health Organization’s essential medicines [1]. Despite the high antitumor activity of DOX against a wide range of malignant neoplasms, its use is associated with serious side effects, such as anaphylaxis; irreversible damage to the endothelium and myocardium, leading to chronic heart failure; and injury to other vital organs (liver and kidneys).

Currently, the molecular mechanisms of DOX’s effects on the cardiovascular system are being actively studied [2], with particular attention being given to the process of vascular remodeling. Among the mechanisms involved in doxorubicin-induced cardiovascular damage, oxidative stress, mitochondrial dysfunction, the initiation of inflammatory processes, endoplasmic reticulum stress, calcium homeostasis disruption, and various forms of cell death (apoptosis, necroptosis, ferroptosis, and pyroptosis), as well as the impaired regulation of autophagy, are being discussed [3,4]. A distinct hypothesis highlights the ability of DOX to irreversibly bind to Topoisomerase 2β (Top2β) in cardiomyocytes, forming a DOX-Top2β-DNA complex. Upon binding to Top2β, DOX inhibits its ability to ligate double-strand DNA breaks, resulting in cell cycle arrest at the G1/G2 phase and the initiation of programmed cell death [5].

In the last 10–15 years, thanks to advancements in gene sequencing technology with a focus on microRNA research, studies have shown the pathological impact of DOX on their expression [6]. MicroRNAs have numerous biological functions and participate in many physiological processes of the cardiovascular system, including differentiation, replication, and regeneration [7]. Research aimed at identifying more precise molecular pathways of DOX’s impact on both cardiomyocytes and endothelial cells will enable the development of the most sensitive and specific diagnostic and treatment methods for anthracycline-induced cardiovascular damage.

To date, the issue of detecting DOX-induced myocardial damage at an early stage remains relevant. Since microRNAs are stable non-coding RNA molecules that can be easily detected in various body fluids and tissues, they hold potential as diagnostic biomarkers and therapeutic targets for preventing cardiovascular pathologies, including DOX-induced cardiomyopathy.

The detection of microRNAs in body fluids and tissues is crucial for understanding their role in gene regulation and disease diagnostics [8]. Common methods include quantitative reverse transcription PCR (qRT-PCR), which provides high sensitivity and specificity for individual miRNAs. Next-generation sequencing enables the comprehensive profiling of miRNAs with high throughput but requires more resources and bioinformatics expertise. Microarray platforms are useful for screening multiple miRNAs simultaneously, offering a cost-effective alternative for large-scale studies. Additionally, techniques like Northern blotting and in situ hybridization are employed for visualizing miRNA expression in tissues. Advanced approaches, such as droplet digital PCR (ddPCR) and nanopore technologies, are emerging for ultra-sensitive detection, even in low-abundance samples like extracellular vesicles or serum.

There are already studies investigating the impact of doxorubicin on microRNAs [6], and they have shown that this chemotherapeutic agent can alter the expressions of various microRNAs involved in the development of cardiovascular diseases. However, despite significant progress in understanding these mechanisms, most existing studies are limited to examining individual microRNAs or specific pathological conditions associated with doxorubicin-induced cardiotoxicity. These studies often do not encompass the full spectrum of microRNAs involved in vascular remodeling and may not thoroughly explore the downstream signaling pathways implicated in this process.

This review provides a detailed examination of the role of microRNAs in vascular remodeling associated with the development of cardiovascular diseases of various etiologies. It summarizes the literature on the modulation of microRNA expression in the context of the pathogenesis of DOX-induced damage, as well as consolidates information on downstream signaling markers activated by microRNAs in response to anthracycline exposure. This study aims to structure and consolidate the current knowledge regarding the role of microRNAs in vascular remodeling during doxorubicin treatment and the development of various cardiovascular diseases, including atherosclerosis, arterial and pulmonary hypertension, aortic aneurysm, vascular stenosis, and tumor neovascularization.

## 2. Article Search and Selection Strategy

The search for published articles and reviews in open-access, peer-reviewed journals was conducted using the following databases: PubMed and Google. Additionally, we used the available article abstracts or full articles on ResearchGate without open access. Most peer-reviewed articles were published in the last 10–15 years, with older works considered as sources of fundamental discoveries. Further database searches were also conducted via Google using the following keywords: doxorubicin, microRNA, biomarker, endothelial-mesenchymal transition, inflammation, fibrosis, endothelium, vascular remodeling, heart disease, and similar. The article and review search was conducted between December 2023 and May 2024.

## 3. MicroRNAs and Their Role in Vascular Remodeling in the Context of Vascular Pathologies of Various Origins

Vascular remodeling is defined as the dynamic process of structural changes in arteries in response to diseases, damaging factors, and aging. The result of this process is the adaptation of blood vessels to current conditions by altering the balance of cell growth, death, and migration, as well as the restructuring of the extracellular matrix [9]. Consequently, vascular remodeling can be considered a hallmark of the most common diseases of our time, such as atherosclerosis, primary hypertension, aortic aneurysm, pulmonary hypertension, and vascular stenosis as a complication of previous angioplasty. Investigating the mechanisms of this process and discovering ways to regulate it could reveal new therapeutic targets for treating these diseases, ultimately reducing the prevalence of cardiovascular pathologies.

MicroRNAs (miRs) are endogenous, small non-coding RNA molecules, typically 22 nucleotides long but ranging from 19 to 25 nucleotides, that play a crucial role in the post-transcriptional regulation of target genes. Currently, the role of microRNAs in the development of many diseases, particularly oncology and cardiovascular pathologies, is being actively studied [10]. Table 1 presents the most studied effects of microRNAs regarding pathological vascular remodeling during the development of vascular diseases of different etiologies.

The miR-221/222 cluster has been shown to play a crucial role in vascular biology by regulating the function of vascular smooth muscle cells and endothelial cells. These microRNAs facilitate vascular remodeling, an adaptive process that involves phenotypic and behavioral changes in vascular cells in response to various types of vascular injury [11]. miR-221/222 stimulate the switch from a “contractile” phenotype of vascular smooth muscle cells to a “synthetic” phenotype, which is associated with the induction of proliferation and mobility, as well as causing their atherogenic calcification [13]. Cordes and colleagues demonstrated that the activities of miR-145 and miR-143 were decreased in injured or atherosclerotic vessels containing proliferating, less differentiated smooth muscle cells [16].

Several studies using mouse models of atherosclerosis and acute coronary syndrome have shown that the increased expression of miR-9 contributes to a reduction in the aortic plaque area, collagen fiber proliferation, macrophage levels, and inflammatory cytokines IL-6, IL-1β, and tumor necrosis factor-α (TNF-α) by suppressing syndecan-2 (SDC2), the FAK/ERK signaling pathway, and p38-mitogen-activated protein kinase (p38MAPK), thereby reducing atherosclerosis processes [20,21]. Additionally, the study by Sysol et al. demonstrated the role of miR-1 in hypoxia-mediated pulmonary vascular remodeling in the context of pulmonary hypertension [60]. It has been shown that the overexpression of miR-150 promotes the AKT/mTOR signaling pathway, inhibits collagen fiber formation, α-SMA, TGF-β1 expression in lung tissues, and PASMCs under hypoxia [42].

Using microarray analysis, Northern blotting, and quantitative real-time polymerase chain reaction methods, Ji and colleagues first demonstrated that the depletion of miR-21 leads to the reduced proliferation of vascular smooth muscle cells and increased cell apoptosis [45]. A 2024 study was the first to show that miR-25-3p directly targets SIRT6. The expression of miR-25-3p leads to the suppression of the Nrf2/ARE and SIRT6 signaling pathways, thereby triggering pathological processes characteristic of intimal hyperplasia, including the proliferation, migration, and phenotypic switching of aberrant vascular smooth muscle cells [23].

**Figure 1:**
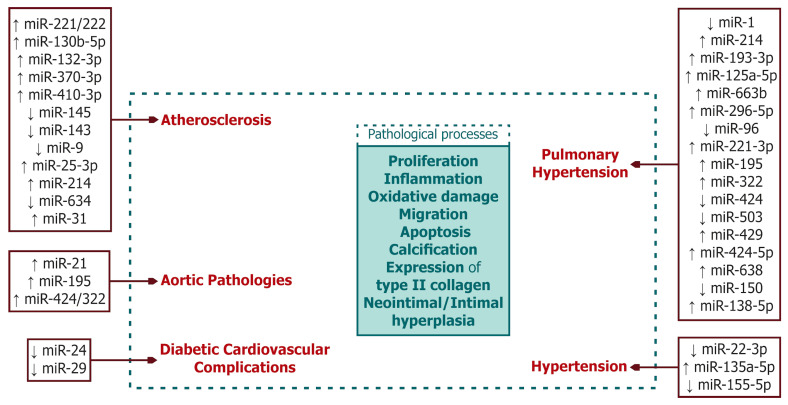
Alterations in the expressions of key known microRNAs during the development of specific cardiovascular pathologies (atherosclerosis, hypertension (arterial and pulmonary), and diabetic cardiovascular complications). ↑, overexpression; ↓, reduced expression.

Figure 1 summarizes information on changes in microRNA expression in cardiovascular diseases.

### MicroRNAs in Tumor Angiogenesis

Exosomes secreted by tumor tissue are known to regulate cell survival, tumor growth, and promote the dissemination of tumor cells. Several studies have demonstrated that microRNAs (miRNAs) are contained and secreted by exosomes, thereby modulating tumor suppressor expression, either enhancing or inhibiting their activity [61].

For example, high levels of miR-21 expression have been observed in hepatocellular carcinoma. miR-21 activates PDK1/Akt signaling in liver cells and PTEN/PI3K/Akt pathways in intestinal cells, which leads to the increased secretion of angiogenic cytokines, including VEGF, MMP2, MMP9, FGF, and TGF-β, and regulates the permeability of tight epithelial junctions [62]. The overexpression of miR-205 and miR-141-3p also promotes tumor angiogenesis via the PTEN/Akt pathway by upregulating VEGFR-2 and NF-kB signaling [63]. Similarly, miR-25-3p and miR-23a modulate the expressions of VEGFR2, ZO-1, occludin, and claudin 5 in endothelial cells, enhancing vascular permeability and angiogenesis [64,65].

Additionally, miR-126 boosts the pro-angiogenic effects of VEGF and FGF, while miR-9 promotes angiogenesis in glioma cells by targeting thrombospondin 2 (THBS-2), patched homolog 1 (PTCH1), and prolyl hydroxylase 3 (PHD3) [66]. Furthermore, miR-142-3p and miR-501-3p contribute to tumor angiogenesis by inhibiting TGF-β receptors 1 and 3 [67]. miR-135b, which is secreted by exosomes, promotes blood vessel growth through FOXO1 inhibition [68]. Other miRNAs, including miR-130a, miR-210, miR-424, the let-7 family, miR-27b, and the miR-17-92 cluster, have also been identified as pro-angiogenic regulators.

Conversely, miR-34a has been shown to negatively regulate tumor angiogenesis by suppressing critical proteins such as E2F3, SIRT1, survivin, and CDK4, directly impairing endothelial cell function. Moreover, the overexpression of miR-34a reduces the levels of proteins involved in VEGF expression, including E2F3, Myc, and c-Met [69].

The contributions of microRNAs to tumor angiogenesis, along with the proposed underlying mechanisms, have been extensively detailed by Zheng et al. [70], Li et al. [71], Hussen et al. [72], Kim et al. [73], and Rupaimoole et al. [74].

Thus, the interactions between microRNAs and their target genes create a complex regulatory network capable of modulating processes such as the proliferation of vascular smooth muscle cells, their migration, apoptosis, and extracellular matrix remodeling, which are crucial in the pathogenesis of several serious cardiovascular diseases. Elucidating the intricate roles of specific microRNAs in cardiovascular pathological remodeling is crucial for advancing medical knowledge. This deeper understanding will pave the way for innovative therapeutic approaches, potentially leading to the development of novel microRNA-based treatments. Ultimately, leveraging the potential of microRNAs can lead to more precise and effective treatments, offering hope for improved outcomes in patients with cardiovascular conditions, including those developed as a result of chemotherapy involving DOX.

## 4. MicroRNA Modulation in Response to Doxorubicin

DOX, a widely used chemotherapeutic agent, exerts its effects through a complex, multifactorial mechanism that affects numerous molecular pathways. Notably, DOX induces a cascade of disruptions, including oxidative stress, inflammatory responses, endoplasmic reticulum stress, and ion homeostasis destabilization. These disruptions, in turn, lead to various forms of cell death, such as apoptosis, necroptosis, ferroptosis, and pyroptosis, while also contributing to the dysregulation of autophagy [75]. MicroRNAs play a key role in regulating these processes by modulating genetic programs that control cellular damage and repair, positioning them as important targets for further investigation in the context of DOX-induced toxicity.

### 4.1. MicroRNA and Apoptosis

**Figure 2:**
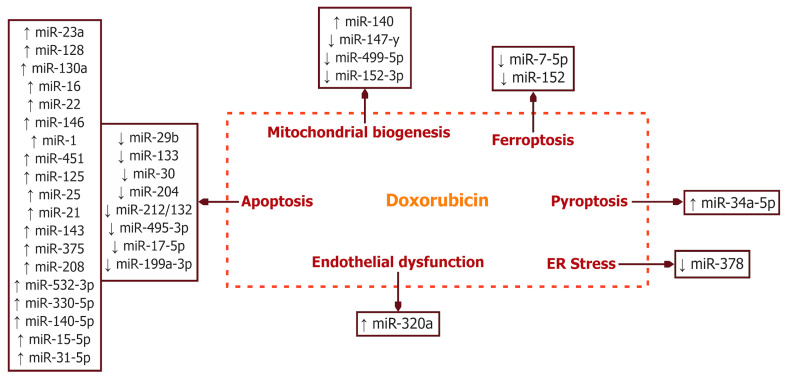
Effects of doxorubicin on microRNA expression in modulating apoptosis, mitochondrial biogenesis, ferroptosis, pyroptosis, endoplasmic reticulum (ER) stress, and endothelial dysfunction. ↑, overexpression; ↓, reduced expression.

To date, apoptosis is the most studied type of cell death induced by DOX [76]. The p53 protein is the main regulator of apoptosis, whose increased levels upon DNA damage induce cell death. MicroRNAs can directly influence p53 or regulate it through downstream targets. Table 2 and Figure 2 summarize the effects of DOX on microRNAs that lead to the initiation of apoptotic processes.

Exposure to DOX induces oxidative stress, leading to elevated miR-23a expression in cells. This upregulation activates p53 protein transcription, forming a complex that directly binds to p53. Consequently, this complex associates with the promoter region of miR-128, indirectly reducing prohibitin expression—an apoptosis inhibitor—thereby initiating cardiomyocyte death [77]. miR-128 is associated with the increased expression of the nuclear protein p65 and the Bax protein, as well as increased caspase-3 activity. These molecules are the main participants in the intrinsic apoptosis pathway. Additionally, the activation of miR-128 expression results in an increase in markers of lipid peroxidation, such as 3,4-Methylenedioxyamphetamine (MDA), 4-hydroxy-2E-nonenal (4-HNE), and 3-nitrotyrosine (3-NT), against the background of decreased activity of superoxide dismutase (SOD), CAT, and glutathione peroxidase (Gpx), leading to cell membrane damage. In the study by Zhang et al., it was shown that miR-128 exerts its effects through the peroxisome proliferator-activated receptor gamma (PPAR-γ), a nuclear hormone receptor that regulates inflammation and proliferation processes, including in the myocardium [79,80]. Thus, the suppression of PPAR-γ expression by miR-128 explains the increase in pro-inflammatory cytokines, lipid peroxidation markers, and the initiation of cardiomyocyte apoptosis. PPAR-γ is also a target for the effects of miR-130a, whose expression increases with DOX treatment [81]. DOX exposure in cardiomyocytes leads to an increase in miR-23a content; however, the realization of its effects is not solely linked to p53. miR-23a inhibits the expression of PGC-1α, which is responsible for mitochondrial biogenesis, and, through increased Drp-1, causes mitochondrial dysfunction, cytochrome c release, caspase-3 activation, and the development of DOX-induced cardiomyopathy [78].

The transcriptional activity of p53 is regulated by wild-type p53-induced phosphatase 1, Wip1, via a feedback mechanism [114]. During DOX treatment, the content of miR-16 in myocardial cells increases, thereby suppressing Wip1 protein expression and consequently activating p53, which leads to the initiation of cardiomyocyte apoptosis [82].

One of the downstream targets of p53 is the protein p21, which, by inhibiting cyclin-dependent kinases, causes cell cycle arrest and leads to cell senescence. High doses of DOX and, consequently, severe oxidative stress activate miR-22, which suppresses the expression of p21 [83]. Research has shown that the main target of miR-22 is SIRT1. Under physiological conditions, SIRT1 suppresses the expression of pro-apoptotic proteins such as p53, p38, p66, and Bax. Additionally, it activates the AMPK and promotes the PGC-1α pathways, thereby supporting cellular, metabolic, and proliferative functions [115,116]. The reduction of SIRT1 levels via miR-22, on the contrary, enhances oxidative stress and triggers cardiomyocyte apoptosis [117]. Moreover, DOX causes a decrease in miR-29b levels and, consequently, an increase in the expression of the pro-apoptotic protein Bax, caspase-3 activity, and the suppression of Bcl-2 effects [85].

In contrast to the aforementioned microRNAs, miR-146 exerts contradictory effects on cardiomyocytes. Under DOX treatment, miR-146 expression increases in cardiomyocytes, which then bind to factor 9b, affecting the stabilizer of p53: the TATA-binding protein (TBP). As a result, p53 inhibition prevents apoptosis processes and activates autophagy, thereby explaining the cardioprotective role of miR-146 [86]. However, other data suggest that miR-146 reduces the expression of the membrane protein from the epidermal growth factor family, ErbB4 [87]. The binding of neuregulin-1 to ErbB4 induces mitogenesis and cell differentiation via the intracellular AKT/mTOR cascade [118]. Therefore, an increase in miR-146 disrupts the processes of differentiation and proliferation of cardiomyocytes, reduces membrane potential, and triggers apoptosis [87].

miR-1 is another regulator of apoptosis. It is actively expressed in myocardial cells and is considered to be a biomarker for the cardiotoxic effects of anthracyclines when myocardial damage is induced by DOX [119]. miR-1 influences anti-apoptotic proteins, primarily Bcl-2, reducing their expression and activating apoptosis processes [88]. It can also be hypothesized that in DOX-induced cardiomyopathy, the association of miR-1 with the genes of heat shock proteins HSP-70 and HSP-60 plays a role, as the regulation of their expression leads to endothelial dysfunction by altering the activity of endothelial nitric oxide synthase [89,90].

During DOX treatment, reduced activity of ARC (apoptosis repressor with caspase recruitment domain) is observed. ARC interacts with caspases 2 and 8 and also inhibits the change in mitochondrial membrane permeability and the release of cytochrome c, thereby preventing apoptosis [120]. miR-532-3p, whose expression level increases with DOX treatment, affects ARC by reducing its activity [91].

There is also a group of microRNAs whose actions are directly aimed at regulating caspases, the proteolytic enzymes involved in apoptotic signaling pathways. The expression level of miR-133 is controlled by the MAPK/ERK pathway. It has been shown that DOX exposure reduces the level of miR-133. This microRNA, by binding to several sites in the caspase-9 transcript, reduces its expression and activity. Thus, a decrease in the amount of miR-133 in myocardial cells leads to an increase in caspase-9 activity, triggering cardiomyocyte apoptosis [92].

Two main signaling pathways regulate the cell cycle: the AMPK pathway and the PI3K/AKT/mTOR signaling pathway. These signaling pathways have antagonistic effects, and the predominance of one of them will determine the cell’s fate. AMPK is activated by increased intracellular calcium levels, LKB1, and AMP/ADP. The activation of this cascade influences multiple targets, leading to the stimulation of autophagy, the inhibition of protein synthesis (through mTOR1 inhibition), cholesterol, and glycogen synthesis while simultaneously enhancing fatty acid catabolism and glucose transport into cells [121]. AKT, in turn, is activated by growth factor receptor tyrosine kinases, inducing mTOR, which promotes increased protein and pyrimidine synthesis and the suppression of autophagy, thereby potentiating cell growth and division [122].

miR-451, miR-25, and miR-125b, which act on the AMPK signaling pathway, exhibit various effects in response to DOX treatment. It has been noted that the level of miR-451 increases in cells treated with DOX. miR-451 directly interacts with Cab39, inhibiting it, which indirectly reduces LKB1 activity [93]. Chen et al. also showed that miR-451 affects the MO25/STRAD/LKB1/AMPK signaling axis by reducing the expression of MO25, an upstream kinase for AMPK [94]. The accumulation of excess autolysosomes in the cell, along with decreased GSH/GSSG and SOD activity, leads to increased ROS and cardiomyocyte damage [93,94]. Additionally, reduced miR-125b expression may be a cause of DOX-induced cardiomyopathy. miR-125b interacts with the protein STARD13, suppressing the nuclear–cytoplasmic translocation of the YES-associated protein (YAP), thereby inducing the AMPK signaling pathway [95].

One of the regulatory proteins of the AMPK and AKT signaling pathways is the dual lipid/protein phosphatase PTEN, a negative regulator of the AKT pathway. Increased PTEN activity regulates genome integrity, cell cycle arrest, and apoptosis induction. DOX exposure has been observed to increase miR-25 levels while reducing PTEN activity [96]. PTEN is a target of miR-25, and its suppression reduces phosphorylated AMPK levels [123] but increases PIP3 levels, initiating the PI3K/AKT/mTOR signaling cascade. As a result, autophagy is inhibited, and protein and pyrimidine synthesis is initiated, promoting cell survival. However, increased miR-25 in the cell is associated with DOX-induced apoptosis, ROS production, and DNA damage [96]. A possible explanation for this contradiction is the temporary activation of AKT due to reduced PTEN activity, followed by inhibition of this pathway [124].

miR-495-3p, miR-17-5p, and miR-21 also influence PTEN activity and effects. miR-495-3p and miR-17-5p have been identified as potential cardioprotective targets in DOX-induced myocardial damage. Studies have shown that DOX reduces the expression of these microRNAs, leading to inhibition of the PI3K/AKT/mTOR pathway and cell apoptosis [97,98]. Additionally, miR-21 increases PTEN expression (Yang et al., 2014) but also exerts its effect through another mechanism. The B-cell translocation gene 2 (BTG2) is involved in cell differentiation, proliferation, and the prevention of apoptosis by inhibiting Akt/Erk and activating glycogen synthase kinase 3β (GSK3β) and cyclophilin D [100]. However, the increase in miR-21 after DOX treatment reduces the expression of this gene and induces cardiomyocyte apoptosis [101].

miR-143 and miR-375 are indirect regulators of the AKT pathway. Under DOX treatment, there is an overexpression of miR-143, which leads to the inhibition of ORP8 and the disruption of AKT phosphorylation [103]. miR-375 also suppresses the cardioprotective effects of the AKT pathway by targeting and inhibiting the PDK1 gene, which encodes a kinase for AKT phosphorylation [102]. miR-375 has non-genomic mechanisms for inducing apoptosis, as it reduces SERCA2a activity, which is affected by DOX, leading to an increase in intracellular calcium [102] and, consequently, activating the endonucleases and caspases responsible for apoptosis. SERCA2a is also a target of miR-330-5p, which additionally exerts inhibitory effects on SIRT6 and BIRC5 [104]. SIRT6 mitigates DOX-induced damage by stimulating endogenous antioxidants and inhibiting p53 transcription [125]. BIRC5 is one of the inhibitors of apoptosis proteins (IAP), suppressing both intrinsic and extrinsic apoptosis pathways by blocking caspases 9, 3, and 7 and the extrinsic pathway protein FADD [126].

As previously shown, some sirtuins are targets of microRNAs. miR-140-5p modulates DOX effects through SIRT2. SIRT2, via forkhead box O3 (FOXO3a) and nuclear factor erythroid 2-related factor 2 (Nrf2), mediates the synthesis of enzymes like superoxide dismutase (SOD), heme oxygenase-1 (HO-1), glutathione-S-transferase (GST), glutamate-cysteine ligase (GCLM), and NADP-oxidoreductase (NQO1), which mitigate oxidative stress. DOX exposure increases miR-140-5p levels, consequently suppressing SIRT2 and Nrf2 [105].

Apoptosis is closely linked to inflammation and autophagy processes. miR-204 regulates all these processes. Increased expression of this microRNA promotes cell survival, inhibits apoptosis and inflammation, and regulates autophagy. However, DOX treatment decreases miR-204 levels, leading to the activation of the nuclear protein HMGB1. Elevated HMGB1 expression suppresses the anti-apoptotic and anti-inflammatory effects of miR-204. Additionally, reduced miR-204 activity is associated with increased levels of TNF-α, IL-1, and IL-6, which are responsible for inflammation, decreased p62 expression, and disrupted LC3II/I ratio, leading to autophagy activation [106].

GATA-4 is a protein that regulates myocardial differentiation and development by controlling the transcription of various genes [127,128]. GATA-4 is targeted by microRNAs such as miR-208a and miR-199a-3p. miR-208a is activated by DOX and suppresses GATA-4 functions, leading to the repression of Bcl-2 and Bcl-xl synthesis and increasing the cell’s pro-apoptotic potential [107]. Conversely, miR-199a-3p activity is suppressed by DOX. miR-199a-3p directly targets GATA-4, activating it and, thus, preventing cardiomyocyte aging and apoptosis [108]. The reduced activity of this microRNA also explains the cardiotoxic effects of DOX. GATA-4 is also indirectly targeted by miR-15-5p. DOX induces an elevation in miR-15-5p levels, leading to the suppression of BMPR1a receptor activity and ALK pathway signaling. Consequently, there is decreased transcription of anti-apoptotic proteins regulated by GATA-4, promoting cardiomyocyte apoptosis [109].

GATA-6, along with GATA-4, is an important transcription factor in cardiomyocytes [129]. DOX activates GATA-6, which inhibits miR-30 expression. Reduced miR-30 levels activate the expression of pro-apoptotic proteins and increase the Bax/Bcl-2 ratio. Additional targets of miR-30 include β1AR and β2AR adrenergic receptors, Giα-2 protein, and BNIP3L/NIX, a mitophagy receptor [111]. Increased β1-AR levels in myocardial cells contribute to chronic sympathetic activation of the heart, triggering apoptosis and myocardial hypertrophy [130], which explains the development of DOX-induced cardiomyopathy. The activity of BNIP3L/NIX, an apoptosis effector [131], increases when miR-30 is inhibited [111]. As a result of miR-30 suppression, p53 expression is activated, which directly binds to the Drp-1 promoter, stimulating its synthesis [110]. miR-30 also affects the level of Beclin 1, a regulator of autophagy [132]. Beclin 1, by binding to Bcl-2, mediates the balance between apoptosis and autophagy [133]. Increased Beclin 1 in cardiomyocytes shifts this balance toward autophagy.

One of the known functions of microRNAs is the regulation of circular RNAs (circRNAs). The main target of miR-31-5p in cardiomyocytes is the protein QKI, which has cardioprotective properties that are mediated by cirPan3. Reduced levels of QKI and cirPan3 by miR-31-5p lead to the activation of apoptosis and myocardial necrosis [112].

Several studies [113,134] have demonstrated the role of the miR-212/132 family in the development of myocardial hypertrophy and autophagy processes. DOX suppresses the expression of miR-212/132, leading to the activation of its apoptotic and atrophic effects through downstream targets such as Fitm2, Rbfox1, Sgk3, and Foxo3 [113].

### 4.2. MicroRNA and Mitochondrial Biogenesis

Mitochondria are essential cellular organelles involved in ATP production, as well as the regulation of apoptosis and the cell cycle. Disruptions in mitochondrial division, fusion, and mitophagy can initiate cell death, making the regulation of mitochondrial biogenesis a critical factor in cell survival, particularly in cardiomyocytes. Table 3 and Figure 2 present the primary effects of DOX on microRNAs that trigger mitochondrial damage.

The DOX-induced downregulation of miR-499-5p results in the upregulation of the p21 [135] and p53 proteins and, through interactions with the α- and β-isoforms of the calcineurin catalytic subunit, contributes to elevated Drp1 levels [136]. The accumulation of these proteins within mitochondria initiates pathological mitochondrial division, ultimately leading to myocardial cell death.

Mitofusin 1 (Mfn1) is a key regulator of mitochondrial biogenesis, facilitating mitochondrial fusion and thereby inhibiting apoptosis by preventing the activation of caspase-3 and caspase-9. MiR-140 binds to the 3′-UTR of Mfn1 mRNA, suppressing Mfn1 expression. Consequently, elevated levels of miR-140 during DOX therapy reduce Mfn1 activity, promoting mitochondrial fission and leading to cardiomyocyte apoptosis [137].

Enhanced mitophagy, which removes damaged mitochondria, is positively correlated with reduced apoptosis. MiR-147-y exerts a cardioprotective effect by promoting mitophagy, directly binding to and inhibiting RAPTOR protein expression, a component of the mTORC1 complex [138]. Inhibiting mTORC1 signaling elevates PINK1 protein levels, which activates PARKIN in response to mitochondrial damage. PARKIN, an E3 ubiquitin ligase, ubiquitinates pro-apoptotic proteins Bax and Bak, thereby preventing apoptosis.

DOX suppresses mitophagy by inducing DNMT1 protein expression through the miR-152-3p/ETS1/RhoH axis. Consequently, DNMT1 upregulation inhibits miR-152-3p and Beclin1 protein expression while activating the ETS1 transcription factor and RhoH protein, a member of the Ras superfamily [139]. This DOX-mediated repression of miR-152-3p activity hinders mitophagy and triggers cardiomyocyte apoptosis.

### 4.3. MicroRNAs and Ferroptosis

Ferroptosis is a novel type of programmed cell death that leads to DOX-induced cardiotoxicity, caused by disruptions in iron metabolism and the induction of ROS formation [140]. The microRNAs involved in the ferroptosis-dependent cardiotoxic effects of DOX are currently not well studied. Table 3 and Figure 2 present the primary effects of DOX on microRNAs that trigger ferroptosis.

The roles of miR-7-5p, miR-152, miR-214, and miR-6852 are suggested in disrupting iron metabolism, leading to the death of cardiomyocytes [141]. The level of miR-7-5p in cells decreases with DOX treatment through the activation of the METTL14/KCNQ1OT1 pathway. Inhibiting miR-7-5p increases the number of transferrin receptors on the cell surface, mediating iron uptake and ROS production, consequently triggering ferroptosis [142]. A potential target through which miR-152 may regulate ferroptosis is the protein Keap1, an inhibitor of NRF2. DOX reduces miR-152 activity, suppressing its inhibitory effect on nuclear factor kappa-light-chain-enhancer of activated B cells (NF-kB) and Keap1. Thus, NF-kB activation stimulates the production of pro-inflammatory cytokines, initiating myocarditis, while Keap1 reduces Nrf2 levels [143], a transcriptional activator of antioxidant proteins, including those inhibiting ferroptosis [144]. There is also evidence of miR-152-dependent regulation of transferrin receptor protein 1 (TfR1) [145]. However, a direct relationship between miR-152 and ferroptosis has not been demonstrated and requires further investigation.

It has been shown that miR-214 can affect proteins such as TfR1, plasmacytoma variant translocation 1 (PVT1), activating transcription factor 4 (ATF4), and p53 [146,147], while miR-6852 is an inducer of ferroptosis in tumor cells through the regulation of cystathionine β-synthase (CBS) expression [148,149]. However, their role in the development of cardiotoxic effects has not been established.

**Table 3:** Effects of doxorubicin on microRNA function in modulating mitochondrial biogenesis, ferroptosis, pyroptosis, ER stress, and endothelial dysfunction.

MicroRNA (↓/↑)	Main Target Proteins	What Does It Lead to?	References
**microRNA and Mitochondrial biogenesis**			
miR-499-5p (↓)	↑ p21 and p53 ↑ Drp1	Activation of mitochondrial division and apoptosis	[135,136]
miR-140 (↑)	↓ Mfn1	Activation of mitochondrial division and apoptosis	[137]
miR-147-y (↓)	↑ mTORC1	Suppression of mitophagy and apoptosis	[138]
miR-152-3p (↓)	↑ ETS1 ↑ RhoH	Suppression of mitophagy and apoptosis	[139]
**microRNA and Ferroptosis**			
miR-7-5p (↓)	↑ TfR1	↑ Fe absorption and ROS production	[142]
miR-152 (↓)	↑ NF-kB and Keap1 ? TfR1	Ferroptosis and inflammation	[141,143]
miR-214 (?)	-	? Ferroptosis	[141]
miR-6852 (?)	-	? Ferroptosis	[141]
**microRNA and ER Stress**			
miR-378 (↓)	↑ PPIA, GRP78, ATF4, and CHOP ↑ LDH ↑ caspase-3	Protein folding disorder and ER stress Dysregulation of oxidative phosphorylation Apoptosis	[150,151,152]
**microRNA and Pyroptosis**			
miR-34a-5p (↑)	↑ NLRP3, IL-18, and GSDMD ↑ Drp1 ↓ MFN2 ↓ SIRT1 and SIRT3	Pyroptosis Disruption of mitochondrial division and fusion Apoptosis	[150,153]
**microRNA and Endothelial dysfunction**			
miR-320a (↑)	↓ VEGF-A ⇒ ↓ eNOS	↓ Microvessel density Reduction of tubule formation Disruption of endotheliocyte migration	[154]

↑, activation; ↓, suppression; ?, expected target protein; ATF4, activating transcription factor 4; CHOP, C/EBP homologous protein; Drp-1, dynamin-related protein 1; eNOS, endothelial nitric oxide synthase; ETS1, E26 transformation-specific 1; GRP78, 78-kDa glucose-regulated protein; GSDMD, Gasdermin D; LDH, lactate dehydrogenase; Mfn1, mitofusion-1; miR, microRNA; mTORC1, mechanistic target of rapamycin complex 1; NF-κB, nuclear factor kappa B; NLRP3, NLR family pyrin domain-containing 3; PPIA, peptidylprolyl isomerase A; RhoH, Ras Homolog Family Member H; SIRT, silent information regulator 2 protein; TfR1, transferrin receptor 1; VEGF-A, vascular endothelial growth factor-A.

### 4.4. MicroRNAs and ER Stress

One of the mechanisms of DOX pathogenesis is endoplasmic reticulum (ER) stress. Table 3 and Figure 2 present the primary effects of DOX on microRNAs that induce ER stress. It has been shown that DOX suppresses the expression of miR-378, which inhibits the hyperactivation of ER stress signaling pathways. Increasing the activity of miR-378 has a cardioprotective effect by regulating three targets [155]: cyclophilin A (PPIA) (modulates protein folding) [152]; LDHA gene (its suppression leads to reduced LDH activity and the regulation of oxidative phosphorylation reactions); caspase-3 (the inhibition of caspase-3 reduces cardiomyocyte apoptosis) [151,156].

### 4.5. MicroRNAs and Pyroptosis

Pyroptosis is a type of programmed cell death characterized by the disruption of the plasma membrane integrity and the rapid release of cellular contents, with key mediators including caspase-1, IL-1β, IL-18, and Gasdermin D (GSDMD). Table 3 and Figure 2 present the primary effects of DOX on microRNAs that initiate pyroptosis.

Recent studies have shown that miR-34a-5p stimulates the process of pyroptosis, contributing to the toxic effects of DOX. The overexpression of miR-34a-5p results in increased levels of the pyroptosis-related proteins NLRP3, IL-18, and GSDMD, while the inhibition of miR-34a-5p significantly reduces their levels. This process of pyroptosis is regulated by mechanisms involving autophagy and mitochondrial biogenesis.

Upon DOX treatment, the level of DRP1, a protein responsible for mitochondrial dysfunction and the initiation of apoptosis, increases, whereas the level of MFN2, which promotes mitochondrial fusion, decreases. Additionally, miR-34a-5p selectively binds to the 3′-UTR of Sirt3 mRNA, inhibiting its expression. DOX-induced suppression of SIRT3 leads to the production of mitochondrial ROS and hampers autophagy processes, thereby activating pyroptosis [153]. In earlier studies, SIRT1 was identified as a target of miR-34a-5p. The suppression of SIRT1 expression resulted in increased levels of p66shc, Bax, and caspase-3 along with decreased activity of Bcl-2 [150].

### 4.6. MicroRNAs and Endothelial Dysfunction

DOX-induced cardiomyopathy is mediated by endothelial dysfunction. In their study, Yin et al. demonstrated the impact of miR-320a on the regulation of new blood vessel formation and the maintenance of vascular homeostasis. Elevated levels of miR-320a were observed in cardiomyocytes treated with DOX. The increase in miR-320a was accompanied by the reduced activity of VEGF-A, resulting in decreased microvessel density, as indicated by the reduced expressions of CD31 and CD34. The suppression of VEGF-A leads to the inhibition of endothelial NO synthase expression, impaired NO release, reduced tube formation, and disrupted endothelial cell migration [154]. These effects promote endothelial dysfunction and the development of DOX’s cardiotoxic effects. Table 3 and Figure 2 present the primary effects of DOX on microRNAs that contribute to endothelial dysfunction.

Thus, DOX, by affecting and altering the expression of microRNAs, leads to the initiation of apoptosis, ferroptosis, pyroptosis, ER stress, and the disruption of mitochondrial biogenesis, thereby causing the death of endothelial and myocardial cells.

## 5. Molecular Markers Involved in Vascular Remodeling in Response to Doxorubicin Exposure

A key process driving vascular remodeling under doxorubicin (DOX) exposure is endothelial–mesenchymal transition (ENDMT), in which endothelial cells lose their characteristic properties and acquire mesenchymal features [157]. This transition is associated with the activation of several molecular markers that regulate inflammatory, fibrotic, and apoptotic pathways [4,158]. Investigating these markers and the mechanisms underlying ENDMT offers deeper insights into the pathogenesis of DOX-associated vascular disorders and may support the development of novel strategies for preventing and treating vascular complications.

Cobb et al. noted that DOX treatment enhances the TGF-β/Smad3 signaling pathway in cardiovascular system remodeling processes [159]. Tsai et al. also demonstrated the crucial role of the TGF-β/Smad signaling pathway in the realization of ENDMT induced by the toxic effects of DOX [160]. Under the action of calcitriol, there was a decrease in Smad2 levels in TGF-β-induced ENDMT in HUVECs. Additionally, vitamin D improved the production of the extracellular matrix by suppressing TGF-β/Smad-mediated ENDMT and the fibroblast-to-myofibroblast transition (FMT), ultimately mitigating fibrosis processes.

Several studies examining tumor cell resistance to DOX-based chemotherapy have revealed that anthracycline affects the expression of microRNAs, including miR-125a-5p [161], miR-150 [162], and miR-424 [163]. These microRNAs have been shown to influence TGF-β levels, playing a role in pathological vascular remodeling associated with the progression of pulmonary hypertension and aortic disease.

Chen and colleagues, in their 2021 study, showed that vitamin D could counteract the reduction in AMPKα and SIRT1 caused by DOX treatment, leading to a decrease in inflammatory processes in endothelial cells [164]. Several studies have shown the role of AMPKα and SIRT1 as key modulators of FOXO3a [165,166]. Using specific inhibitors of SIRT1 and AMPKα, the authors established that vitamin D-mediated IL-10 expression, an anti-inflammatory cytokine, occurs through the post-translational activity of FOXO3a via the SIRT1/AMPKα signaling pathway [164,167]. Therefore, the AMPKα/SIRT1/FOXO3a pathway is also involved in the realization of the toxic effects of DOX on endothelial cells.

In a model of 7–8-week-old C57Bl/6 mice treated with DOX, elevated ALK4/5 ligands, including TGF-β2 and activins, were identified, leading to the suppression of proliferation of epithelial-origin cells and the remodeling of the cardiovascular system [168].

The excessive accumulation of ROS due to DOX exposure leads to the activation of the NF-κB/Snail signaling pathway, which initiates ENDMT processes and disrupts autophagy regulation [169]. Ismail and his colleagues recently determined that mangiferin (MGN), a xanthone and C-glucosylxanthone derivative, significantly reduces the levels of inflammatory cytokines (COX-2 and TNFα) in HUVECs treated with DOX [170]. Thus, MGN protects against endothelial dysfunction in vitro due to its potent anti-inflammatory action. Moreover, MGN prevents DOX-induced NF-κB translocation and its binding to DNA, thereby reducing its pro-apoptotic role in endothelial cells.

The study confirmed the importance of the anti-apoptotic PI3K/AKT pathway, which is involved in controlling cell growth, proliferation, survival, and migration [171]. The PI3K/AKT signaling pathway initiates with extracellular oxidative stress triggering the activation of a cell surface tyrosine kinase. This activation of PI3K stimulates an anti-apoptotic kinase, leading to the phosphorylation of Nrf2, which is released from its suppressor gene Keap1. Subsequently, Nrf2 translocates to the nucleus, where it enhances the production of phase II antioxidant enzymes. DOX reduces the levels of PI3K/AKT and Nrf2 proteins, while mangiferin treatment of HUVECs activates Nrf2 via the PI3K/AKT and ERK pathways, preventing endothelial damage [170].

Aging of endothelial cells contributes to chronic inflammation and the development of endothelial dysfunction, as aging cells acquire a pro-inflammatory secretory phenotype. In a 2022 study, Graziani et al. demonstrated that in isolated rat heart microvascular endothelial cells, DOX has a long-lasting, irreversible effect, leading to endothelial cell aging and the disruption of protein synthesis by reducing VEGFR2 levels [172]. DOX can regulate the mTOR pathway, thereby inducing p53 activation, which, through a feedback mechanism, inhibits mTOR signaling [173]. mTOR, in turn, can suppress angiogenesis by reducing VEGF/VEGFR2 levels [174,175]. Lorenzo et al. showed that DOX causes significant accumulation of p53, induces CD95 gene expression, and initiates apoptosis in HUVECs [176]. Additionally, it was found that the activity of NADPH oxidase 2 (Nox2), which is altered by DOX treatment, also affects endothelial cell aging by intensifying oxidative stress processes [177]. Shamoon and colleagues recently identified another key player in initiating inflammatory processes in endothelial cells. They showed that DOX induces the expression of NLRP3, a key factor in the innate immune system that is capable of stimulating the release of IL-1β [178].

Recent studies have also focused on examining the resistance of various tumor cells, predominantly breast cancer cells, to DOX, revealing modulations in the expressions of microRNAs such as miR-221 [179], miR-222 [180], miR-1 [119], miR-21 [181], miR-24 [182], and miR-150 [162]. In vascular system pathologies, the dysregulation of these microRNAs has been shown to inhibit the PTEN/PI3K/AKT signaling pathway, activate the AKT/mTOR pathway, and induce pro-inflammatory factors such as NF-κB, TNFα, IL-1β, and IL-18 (Table 1). Further investigation is needed to elucidate the roles of these known microRNAs in vascular remodeling associated with atherosclerosis, arterial and pulmonary hypertension, and aortic disease, particularly in connection with DOX, as they may also serve as potential targets in developing therapeutic strategies for anthracycline-induced ENDMT.

A recent study first discovered that DOX can cause DNA damage in endothelial cells, leading to the release of double-stranded DNA from the nucleus and mitochondria into the cytosol. The cytosolic double-stranded DNA then binds to cyclic guanosine monophosphate–adenosine monophosphate synthase (cGAS), activating it, which results in the production of cyclic GMP-AMP. This cyclic GMP-AMP induces STING, subsequently activating TANK-binding kinase 1 and interferon regulatory factor 3. Interferon regulatory factor 3 then enters the nucleus and binds to the CD38 promoter, stimulating its expression. This causes a decrease in NAD+ levels in myocardial endothelial cells, leading to mitochondrial dysfunction, inflammation, endothelial dysfunction, the rarefaction of cardiac capillaries, and reduced cardiac perfusion [183].

It is known that the viability of endothelial cells is regulated by MAPKs—ERK1/2, JNK, P38, and ERK5 [184]—whose activation provides a positive environment for VEGFR2 function and induction of angiogenesis.

Multiple studies conducted by Wilkinson and colleagues have demonstrated that DOX can disrupt the barrier function of cardiac and dermal microvascular endothelial cells. This disruption involves the loss of ZO-1 from tight junctions, which occurs through the dysregulation of ERK5 and results in heightened paracellular permeability [185,186]. Similar findings were also reported by Fernandez-Fernandez et al. [187]. Studies conducted on rats demonstrated that DOX exposure resulted in altered permeability of cardiac microvascular endothelial cells in vivo, correlating with decreased left ventricular function. Additionally, Zhang and colleagues in 2011 established that DOX can suppress the cardiac expression of Cx43 and Cx45 via the activation of the JNK signaling pathway, leading to the loss of Cx43/Cx45 gap junctions and subsequent spatial remodeling of gap junctions, ultimately accompanied by the progressive development of DOX-induced myocardial damage [188]. Several researchers have identified the presence of Cx43 in cardiac mitochondria [189,190,191]. Pecoraro and colleagues demonstrated that DOX disrupts Ca2+ homeostasis, which is evident even after a single administration of this chemotherapeutic agent, and affects the expression and localization of Cx43 [192].

In a 2020 study by Jahn et al., it was found that endothelial progenitor cells exhibit greater sensitivity to DOX compared to embryonic stem cells differentiated into endothelial-like and cardiomyocyte-like cells [193].

**Figure 3:**
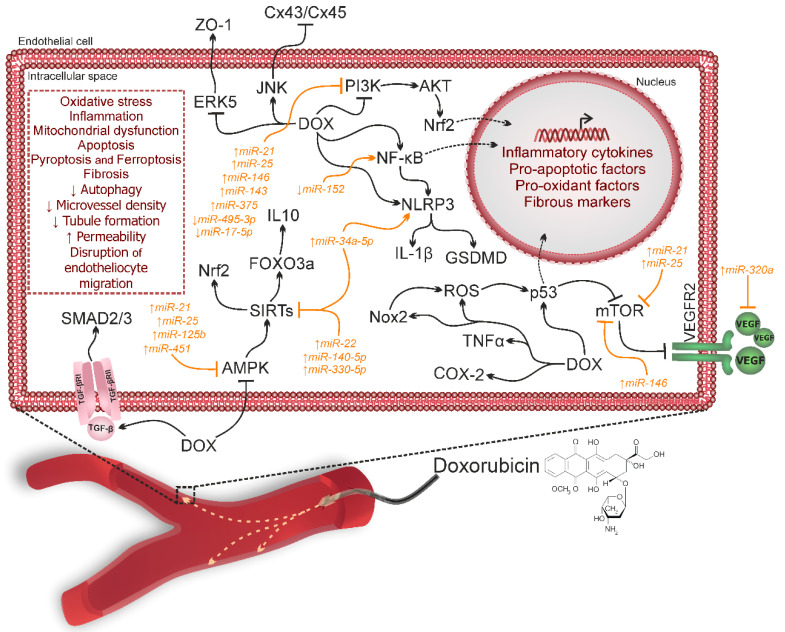
Key signaling pathways involved and their effects in vascular remodeling under doxorubicin exposure. ↑, activation; ↓, suppression; AKT, protein kinase B; AMPK, 5′ AMP-activated protein kinase; COX-2, cyclooxygenase-2; Cx43/Cx45, connexin 43/45; DOX, doxorubicin; ERK5, extracellular signal-regulated kinase 5; FOXO3a, forkhead box protein O3a; GSDMD, Gasdermin D; IL10, interleukin-10; JNK, c-Jun N-terminal kinases; mTOR, mammalian target of rapamycin; NF-κB, nuclear factor kappa-light-chain-enhancer of activated B cells; Nrf2, nuclear factor erythroid 2-related factor 2; PI3K, phosphoinositide 3-kinases; ROS, reactive oxygen species; SIRT, silent information regulator 2 protein; TGF-β, transforming growth factor beta; TNF-α, tumor necrosis factor alpha; VEGFR, vascular endothelial growth factor receptor; ZO-1, zonula occludens-1.

Thus, the administration of DOX exerts a detrimental effect on the vascular system through ENDMT, which is triggered by the activation of key signaling pathways such as TGF-β/Smad, NF-κB/Snail, mTOR/VEGF, cGAS/STING/CD38, ERK5, and JNK. DOX has been shown to induce oxidative stress, inflammation, and apoptosis in endothelial cells, ultimately leading to endothelial dysfunction, vascular remodeling, and fibrosis. Moreover, the microRNAs involved in vascular remodeling in other vascular system pathologies may also initiate ENDMT processes in response to DOX exposure. Figure 3 presents a schematic overview of the signaling pathways involved in ENDMT that mediate DOX-induced endothelial damage, as well as the key microRNAs whose expressions are modulated in response to anthracycline treatment.

## 6. Discussion

Over the past 10-15 years, the study of microRNAs’ role in pathological vascular remodeling has become a key area of focus, holding significant importance for a deeper understanding of cardiovascular diseases and the development of new therapeutic strategies. To date, DOX remains a widely used chemotherapeutic agent for treating various types of cancer. However, its application is associated with significant cardiotoxic effects (decreased left ventricular function, cardiomyopathy, arrhythmias, pericarditis and myocarditis, and acute heart failure), often determining the impossibility of continuing chemotherapy or necessitating a reduction in the dose of the chemotherapeutic drug. Increasingly, evidence shows that DOX also exerts damaging effects on the vascular endothelium, which, in turn, contributes to the development of cardiotoxic effects [194,195].

A relatively new area of interest is studying the impact of DOX on microRNAs in the context of vascular remodeling. Vascular remodeling is a multi-level process involving metabolic, structural, and genetic changes aimed at adapting the vascular system to new conditions.

At the metabolic level, changes occur in the energy metabolism of vascular wall cells. For example, the consumption and utilization of glucose and oxygen may alter, leading to changes in the production of ATP and other metabolites. These changes can influence the activities of enzymes and signaling molecules that regulate cell growth, proliferation, and migration.

Structurally, remodeling involves changes in the composition and organization of the extracellular matrix, alterations in the thickness of the vascular wall, and changes in the diameter of the vessels. There is a reorganization of the cellular cytoskeleton, which can lead to changes in the shape and motility of endothelial and smooth muscle cells. These processes are often accompanied by an increase or decrease in the number of vessels, altering the overall vascularization of tissues.

Genetically, remodeling includes changes in the expression of genes coding for proteins involved in the structural and functional reorganization of the vessels. These may include the genes responsible for the synthesis of collagen and elastin and enzymes involved in the metabolism of the extracellular matrix, as well as genes regulating the cell cycle, apoptosis, and autophagy. Changes in the expression levels of these genes can result from epigenetic modifications such as DNA methylation and histone modification, as well as the influence of various signaling pathways and transcription factors.

Upon intravenous administration, the chemotherapeutic agent penetrates endothelial cells actively, inducing oxidative and nitrosative stress. This triggers the activation of profibrotic, pro-inflammatory, and pro-apoptotic signaling pathways such as TGF-β/Smad, NF-κB, and COX-2/TNFα. Concurrently, it inhibits anti-apoptotic and angiogenesis-stimulating markers, including AMPKα/SIRT1/FOXO3a, VEGF/VEGFR2, and PI3K/AKT/Nrf2. These changes lead to the reprogramming of the genetic program of endothelial cells and trigger mechanisms responsible for vascular remodeling.

The activation or inhibition of the aforementioned signaling pathways can be directly influenced by microRNAs, whose expression is modulated in response to DOX exposure. For instance, elevated levels of miR-22, miR-140-5p, miR-330-5p, miR-21, miR-25, miR-125b, miR-451, and miR-34a-5p lead to the suppression of the AMPKa/SIRT pathway. The overexpression of miR-21, miR-25, miR-146, miR-143, and miR-375, alongside reduced levels of miR-495-3p and miR-17-5p, decreases PI3K/AKT and Nrf2 protein levels through PTEN upregulation. Additionally, miR-21, miR-25, and miR-146 activation under DOX exposure stimulates p53 and inhibits the mTOR signaling pathway (Figure 3).

Potential microRNA candidates that may be impacted by DOX in the context of vascular remodeling include miR-1, miR-125a-5p, miR-663b, miR-296-5p, miR-150, miR-138-5p, miR-195, miR-424, miR-322, miR-410-3p, miR-221/222, miR-24, miR-9, miR-126, miR-25-3p, miR-214, and miR-221-3p. Changes in the expression of these microRNAs have been reported in other vascular pathologies (Table 1).

Currently, it is known that several of these microRNAs play key roles in both vascular remodeling and tumor angiogenesis, although their functions may vary depending on the context. For instance, miR-126 promotes angiogenesis in both ischemic tissues and tumors by enhancing VEGF signaling, yet in tumors, it can also act as a tumor suppressor by inhibiting cancer cell proliferation. miR-21, a pro-angiogenic microRNA, promotes vascular smooth muscle cells proliferation in vascular diseases and enhances tumor angiogenesis through the activation of VEGF and MMP, while its role in inflammation links it to both pathological vascular remodeling and the tumor microenvironment. miR-143/145 typically suppress vascular smooth muscle cells proliferation and angiogenesis, contributing to abnormal vascular growth and cell migration in both vascular disorders and tumors. In some cases, miR-25 supports endothelial cell survival and reduces oxidative stress, while in others, it promotes ENDMT. Simultaneously, miR-25 enhances tumor angiogenesis by stabilizing HIF-1a and increasing VEGF expression. miR-9 induces inflammation and proliferation in both vascular diseases and tumors, promoting VEGF expression and advancing ENDMT processes.

**Figure 4:**
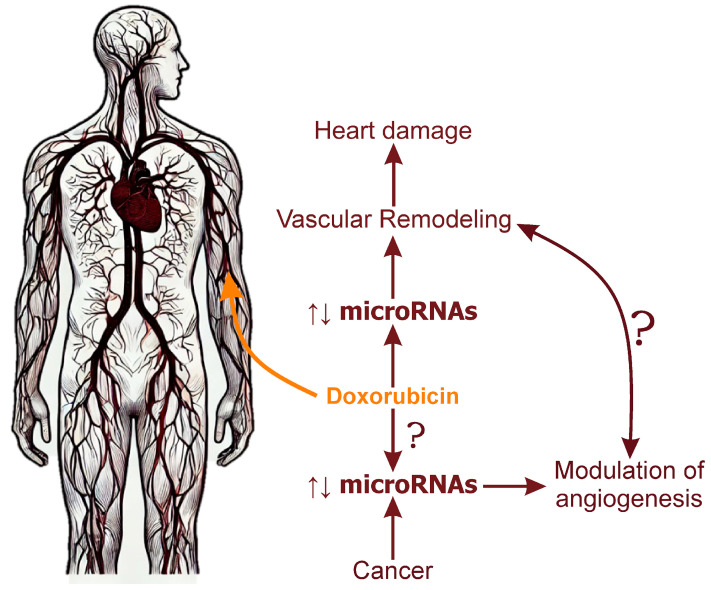
The impact of doxorubicin on microRNA expression in vascular remodeling and tumor angiogenesis. ↑, activation; ↓, suppression.

It is important to recognize that although DOX is intended to reduce tumor vascularization by damaging endothelial cells and inhibiting pro-angiogenic signals, changes in microRNA profiles in response to chemotherapy can produce opposite effects (Figure 4). For example, miR-9 is often upregulated in tumor cells following DOX treatment. The pro-angiogenic effects of miR-9, facilitated by the downregulation of antiangiogenic factors such as thrombospondin-1 and E-cadherin, enable angiogenesis to continue even in the presence of DOX [196,197]. Additionally, miR-9 promotes the ENDMT and increases VEGF expression, further driving angiogenesis under therapeutic pressure.

In certain cancer models, miR-25 has been shown to stimulate angiogenesis by targeting prolyl hydroxylase domain protein 1 (PHD1), a negative regulator of HIF-1α. Under DOX treatment, elevated miR-25 levels may help stabilize HIF-1α, leading to increased VEGF expression [198] and enhanced angiogenesis in hypoxic conditions, which are common in tumors undergoing chemotherapy. This effect may contribute to tumor survival and growth, underscoring the complexity of microRNA-mediated angiogenic responses under DOX exposure. These mechanisms require further experimental investigation.

Further investigation into the mechanisms by which DOX alters microRNA expression and their downstream effects on signaling pathways is essential. Integrating multi-omics approaches, including transcriptomics, proteomics, and epigenomics, could provide a holistic understanding of how DOX influences vascular remodeling and tumor biology. Such studies would identify novel microRNA targets and clarify their dual roles in disease progression and recovery. By bridging the gap between basic science and clinical application, future efforts can pave the way for innovative, multi-targeted approaches that enhance therapeutic efficacy while reducing adverse effects.

The authors do not examine in depth how the aforementioned microRNAs (Section 3, Section 4 and Section 5) might be employed as potential markers for assessing the risk of DOX-induced vascular remodeling, which may be a limitation of this work. Researchers such as Kuang et al. [6], Skála et al. [199], Zangouei et al. [200], Rosenfeld et al. [201], and Tutunchi et al. [202] address this issue in greater detail in their studies.

Therefore, the dysregulation of microRNAs in both vascular pathologies and tumor progression illustrates their dual role in either promoting disease advancement or facilitating recovery, depending on the context. A deeper understanding of the specific roles of microRNAs in mediating DOX’s effects on the vascular system and tumor microenvironment could provide valuable insights into developing therapeutic strategies that enhance the efficacy of chemotherapy while minimizing cardiovascular toxicity and tumor resistance.

## 7. Conclusions

Over the past decade, the exploration of microRNAs’ roles in pathological vascular remodeling has emerged as a critical area of research, shedding light on the complexities of cardiovascular diseases and their interplay with cancer therapies. DOX remains a cornerstone in cancer treatment but is associated with substantial cardiovascular toxicity, including effects on the vascular endothelium that exacerbate cardiotoxicity.

DOX-induced vascular remodeling is a multifaceted process involving metabolic, structural, and genetic changes in vascular tissues. These alterations are driven by oxidative stress and dysregulated signaling pathways, which microRNAs modulate. The differential expression of microRNAs, such as miR-21, miR-25, miR-146, and miR-9, significantly influences key pathways like TGF-β/Smad, PI3K/AKT, and VEGF/VEGFR2, thereby affecting endothelial cell function, angiogenesis, and tumor progression.

The dual role of microRNAs in vascular remodeling and tumor angiogenesis highlights their potential as therapeutic targets. While some microRNAs enhance angiogenesis and tumor survival under DOX treatment, others exhibit protective effects against vascular damage. Understanding these context-dependent roles offers an opportunity to optimize chemotherapy outcomes by mitigating cardiovascular risks and overcoming tumor resistance.

Future research should focus on identifying specific microRNAs as biomarkers for DOX-induced vascular remodeling and investigating therapeutic strategies that modulate their expression. Such insights could pave the way for innovative approaches to reduce DOX-associated toxicity and improve patient outcomes in cancer therapy.

## Figures and Tables

**Table 1:** Effects of microRNAs in pathological vascular remodeling during the development of vascular diseases of different etiologies.

Vascular Diseases	Experimental Model	MicroRNA (↓/↑)	Main Target Proteins	What Does It Lead to?	References
Atherosclerosis and smooth muscle cell phenotypic modulation	Hypertension, obesity, metabolic syndrome, coronary heart disease, and carotid atherosclerosis	miR-221/222 (↑)	↓ C-kit ↓ p27/Kip1 ↓ ICAM1 ↓ VCAM1 ↓ eNOS ↓ Est1	↓ proliferation ↓ migration ↑ neointimal hyperplasia	[11,12]
		(↓) in neglected plaques	↓ RASA1 ↓ PTEN ↓ C-kit ↓ p21/Cip1 ↓ p27/Kip1 ↓ p57/Kip2 ↓ PUMA	↑ proliferation ↑ migration ↑ dedifferentiation ↓ apoptosis ↑ atherogenic calcification	[13]
	Obesity	miR-221-3p (↑)	↑ PGC-1α	↑ proliferation ↑ migration	[14]
	Injured or atherosclerotic vessels	miR-143/145 (↓)	↓ myocardin ↓ KLF4, 5 ↑ Elk-1 ↓ Jag-1/Notch	↑ proliferation ↓ differentiation	[15,16]
	Balloon-induced injury procedure in carotid artery	miR-132-3p (↑)	↑ p21 and p27 SOCS2 (?)	↑ proliferation	[17]
		miR-370-3p (↑)	↑ p21 and p27 ↓ BMP-7 ⇒ ↓ phosphorylation of SMAD1/5/9	↑ proliferation	
		miR-410-3p (↑)	SMAD6 (?)	↑ inflammation	
		miR-130b-5p (↑)	TSPAN2 (?)	↑ inflammation ↑ proliferation of VSMCs ↓ proliferation of ECs	
		miR-146a (↑)	↓ KLF4	↑ proliferation of VSMCs ↑ migration ↑ neointima formation	[18]
		miR-200c (↓)	↓ KLF4	↑ proliferation	[19]
	Atherosclerosis/acute coronary syndrome	miR-9 (↓)	↑ SDC2 ↓ KLF5	↑ inflammation ↑ proliferation ↑ migration	[20,21,22]
	-	miR-25-3p (↑)	SIRT6 ⇒ ↓ Nrf2/ARE	↑ intimal hyperplasia ↑ proliferation ↑ migration ↑ phenotypic switch of VSMCs	[23]
	Angiotensin II-induced hypertension	miR-214 (↑)	↑ Smad7 ↓ phosphorylation of Smad3	↑ proliferation ↑ migration ↑ inflammation of VSMCs	[24]
		miR-634 (↓)	↑ Wnt4/β-catenin	↑ proliferation ↑ migration	[25]
	Coronary heart disease	miR-31 (↑)	↑ LATS2 ↑ PCNA ↓ CREG	↑ proliferation	[26,27]
Pulmonary hypertension	Pulmonary arterial hypertension	miR-1 (↓)	↑ SphK1/S1P ⇒ ↑ Notch3 ↑ ET-1 ⇒ ↑ NF-κB	↑ proliferation and migration of PASMCs	[28,29]
		miR-214 (↑)	↓ MEF2C–myocardin–leiomodin1	Inhibition of the “contractile” phenotype	[30]
		miR-193-3p (↑)	↓ PAK4 ⇒ ↓ p-AKT/AKT	Weakens vascular remodeling	[31]
		miR-125a-5p (↑)	↓ TGF-β1 and IL-6 ⇒ ↓ STAT3 and Smad2/3	↓ proliferation of PASMCs ↑ apoptosis of PASMCs	[32]
		miR-663b (↑)	↓ AMPK/SIRT1	↑ proliferation, inflammation, oxidative stress, and migration of PASMCs	[33]
		miR-296-5p (↑)	↑ TGF-β1/p38 MAPK	↑ proliferation of PASMCs	[34]
		miR-96 (↓)	↑ BMP-4 ⇒ ↓ TRB3 ⇒ ↑ NF-κB	↑ apoptosis of PASMCs ↑ transformation of PASMCs into an undifferentiated phenotype	[35]
		miR-221-3p (↑)	↓ AXIN2	↑ proliferation of PASMCs	[36]
		miR-322 (↑)	↑ HIF-1α ↓ BMP-Smad	↑ proliferation ↑ migration	[37]
		miR-424 (↑)	↓ CUL2 ⇒ destabilization of the E3 ligase assembly ⇒ ↑ HIF-1α		[38]
		miR-424 (↓)	↑ FGF2/FGFR1	↑ proliferation of PASMCs	[39]
		miR-503 (↓)			
		miR-429 (↑)	↓ CaSR ⇒ ↓ Ca2+ inflow	↓ proliferation of PASMCs	[40]
		miR-424-5p (↑)			
		miR-638 (↑)	↑ NR4A3/cyclin D1	↑ proliferation of PASMCs	[41]
		miR-150 (↓)	↑ TGF-β1 ↑ α-SMA ↑ AKT/mTOR	↑ formation of collagen fibers ↑ proliferation of PASMCs	[42]
		miR-138-5p (↑)	↓ SIRT1 ↑ Mst1	↑ proliferation of PASMCs ↑ migration of PASMCs ↑ apoptosis	[43,44]
Aortic pathologies	Abdominal aortic aneurysm	miR-21 (↑)	↓ PTEN ⇒ ↑ AKT ↑ Bcl-2 ↑BMP-4 ⇒ ↓ DOCK 4, 5, 7 ⇒ ↑ small GTFase	↑ proliferation ↓ apoptosis ↓ migration properties and organization of the cytoskeleton	[45,46,47,48]
		miR-195 (↑)	↓ Smad3	↓ proliferation ↑ apoptosis ↑ expression of type II collagen	[49]
		miR-424/322 (↑)	↓ Smad2,3/RUNX2	↓ proliferation ↑ apoptosis	[50]
Hypertension	Spontaneously hypertensive rat	miR-22-3p (↓)	↑ CHD9	↑ remodeling ↑ oxidative stress ↓ switching PASMCs from a “synthetic” phenotype to a “contractile” phenotype	[51]
		miR-135a-5p (↑)	↓ FNDC5	↑ proliferation of VSMCs	[52]
		miR-155-5p (↓)	↑ BACH1	↑ migration ↑ oxidative damage of ECs	[53]
Inflammation and diabetic cardiovascular complications	Model of diabetes (high-fat and high-sugar diet)	miR-24 (↓)	↑ NLRP3 ↑ ASC/PYCARD ↑ caspase-1 ↑ CD45 ↑ IL-1β and IL-18 ↑ TNF-α ↑ AKT/mTOR ↑ JNK1/2, ERK1/2, RAS, PDGF-R, AP-1, P27, and PCNA ↓ CD31 ↓ SMA-α	↑ neointimal hyperplasia ↑ proliferation ↑ migration ↓ rate of re-endothelization	[54,55,56]
	miR-29 KO mice Type 2 diabetes	miR-29 (↓)	↑ PGC1α ↑ COL1A1 ↑ MMP2	↑ aortic stiffness Changes in mitochondrial genesis	[57,58,59]

↑, activation; ↓, suppression; ?, expected target protein; AKT, Akt kinase; AMPK, adenosine monophosphate-activated kinase; AP-1, activating protein-1; ASMCs, aortic smooth muscle cells; AXIN2, axis inhibition protein 2; BACH1, BTB domain and CNC homolog 1; Bcl-2, B-cell leukemia/lymphoma 2; BMP, bone morphogenetic protein; C-kit, KIT proto-oncogene, receptor tyrosine kinase; CaSR, calcium-sensing receptor; CD, cluster designation; CHD9, Chromodomain Helicase DNA Binding Protein 9; COL1A1, collagen type I alpha 1 chain; CREG, Cellular Repressor of E1A-stimulated Genes; CUL2, Cullin-2; DOCK, dedicator of cytokinesis protein; ECs, endothelial cells; Elk, ETS-domain transcription factor; eNOS, endothelial nitric oxide synthase; ERK, extracellular signal-regulated kinase; Est1, SULT1E1, sulfotransferase family 1E member 1; ET-1, endothelin 1; FGF, fibroblast growth factor; FGFR1, fibroblast growth factor receptor 1; FNDC5, fibronectin type III domain-containing protein 5; HIF-1α, hypoxia-inducible factor 1-alpha; ICAM, Inter-Cellular Adhesion Molecule 1; IL, interleukin; Jag-1, jagged canonical Notch ligand 1; JNK1/2, c-jun-N-terminal kinase 1/2; KLF, Krüppel-like transcription factor; LATS2, large tumor suppressor kinase 2; LMOD1, leiomodin1; MAPK, mitogen-activated protein kinase; MEF2C, myocyte enhancer factor 2C; miR, microRNA; MMP2, matrix metallopeptidase 2; Mst1, macrophage stimulating 1; mTOR, mammalian target of rapamycin; MYOCD, myocardin; NF-κB, nuclear factor kappa B; NLRP3, NLR family pyrin domain containing 3; Notch, Notch signal pathway; NR4A3, nuclear receptor 4A3; Nrf2, nuclear factor erythroid 2-related factor 2; p-AKT, phosphorylated AKT; p21/Cip1, cyclin-dependent kinase inhibitor 1A; p57/Kip2, cyclin-dependent kinase inhibitor 1C; PAK4, p21 activated kinase 4; PASMCs, pulmonary artery smooth muscle cells; PCNA, proliferating cell nuclear antigen; PDGF-R, platelet-derived growth factor receptor; PGC-1α, peroxisome proliferator-activated receptor gamma coactivator 1-alpha; PTEN, phosphatase and tensin homolog; PUMA, p53 upregulated modulator of apoptosis; PYCARD, apoptosis-associated speck-like protein containing a CARD; RASA1, RAS P21 protein Activator 1; RUNX2, Runt-related transcription factor 2; S1P, sphingosine-1-phosphate; SDC2, syndecan 2; SHR, spontaneously hypertensive rats; SIRT, silent information regulator 2 protein; SMCs, smooth muscle cells; SOCS2, suppressor of cytokine signaling 2; SphK1, sphingosine kinase 1; STAT, signal transducer and activator of transcription; TGF-β1, transforming growth factor beta 1; TNF-α, tumor necrosis factor α; TRB3, Tribbles-like protein 3; TSPAN2, tetraspanin 2; VCAM, vascular cell adhesion molecule; VSMCs, vascular smooth muscle cells; α-SMA, alpha-smooth muscle actin.

**Table 2:** Effects of doxorubicin on microRNA function, leading to the activation of apoptotic processes.

MicroRNA (↓/↑)	Main Target Proteins	What Does It Lead to?	References
**microRNA and Apoptosis**			
miR-23a (↑)	↑ p53 transcription ↓ PGC-1a (↑ Drp-1)	Activation of the intrinsic pathway of apoptosis and mitochondrial dysfunction	[77,78]
miR-128 (↑)	↓ prohibitin ↓ PPAR-γ	Activation of the intrinsic pathway of apoptosis, lipid peroxidation, and inflammation	[77,79,80]
miR-130a (↑)	↓ PPAR-γ	Activation of the intrinsic pathway of apoptosis, lipid peroxidation, and inflammation	[81]
miR-16 (↑)	↓ Wip1 ⇒ ↑ p53	Activation of the intrinsic pathway of apoptosis	[82]
miR-22 (↑)	↓ p21 ↓ SIRT1	Activation of the intrinsic pathway of apoptosis	[83,84]
miR-29b (↓)	↑ Bax and caspase-3 ↓ Bcl-2	Activation of the intrinsic pathway of apoptosis	[85]
miR-146 (↑)	↓ p53 via TBP	Cardioprotection	[86]
	↓ ErbB4 ⇒ ↓ AKT/mTOR	Reduced proliferation and differentiation, leading to the initiation of apoptosis	[87]
miR-1 (↑)	↓ Bcl-2 Changing of eNOS	Activation of the intrinsic pathway of apoptosis and endothelial dysfunction	[88,89,90]
miR-532-3p (↑)	↓ ARC	Activation of apoptosis	[91]
miR-133 (↓)	↓ caspase-9	Cardioprotection	[92]
miR-451 (↑)	↓ LKB1/AMPK	Accumulation of autolysosomes and ROS production	[93,94]
miR-125b (↑)	↓ YES1 ⇒ ↑ AMPK/p53	Apoptosis and stopping the cell cycle	[95]
miR-25 (↑)	↓ PTEN ⇒ ↓ AMPK ↑ PI3K/AKT/mTOR	Apoptosis and the inhibition of autophagy	[96]
miR-495-3p (↓)	↑ PTEN ⇒ ↓ AKT	Apoptosis and stopping the cell cycle	[97]
miR-17-5p (↓)	↑ PTEN ⇒ ↓ AKT	Apoptosis and stopping the cell cycle	[98]
miR-21 (↑)	↑ PTEN ⇒ ↓ AKT ↓ BTG2 ⇒ ↑ Akt/Erk	Apoptosis and stopping the cell cycle	[99,100,101]
miR-143 (↑)	↓ ORP8 ⇒ ↓ AKT	Apoptosis and stopping the cell cycle	[102,103]
miR-375 (↑)	↓ PDK1 ⇒ ↓ AKT ↓ SERCA2a	Apoptosis, stopping the cell cycle, and the accumulation of intracellular calcium	[102]
miR-330-5p (↑)	↓ SERCA2a ↓ SIRT6 and BIRC5	Apoptosis and the accumulation of intracellular calcium	[104]
miR-140-5p (↑)	↓ SIRT6 ⇒ ↓ Nrf2, FOXO3a	Oxidative stress	[105]
miR-204 (↓)	↑ HMGB1	Apoptosis, inflammation, and autophagy	[106]
miR-208a (↑)	↓ GATA-4	Apoptosis	[107]
miR-199a-3p (↓)	↓ GATA-4	Apoptosis	[108]
miR-15-5p (↑)	↓ BMPR1a ⇒ ↓ ALK ⇒ ↓ GATA-4	Apoptosis	[109]
miR-30 (↓)	↑ Bax/Bcl-2 and p53 ↑ β1-AR ↑ Beclin 1	Apoptosis, myocardial hypertrophy, and autophagy	[110,111]
miR-31-5p (↑)	↓ QKI ⇒ ↓cirPan3	Apoptosis and necrosis	[112]
miR-212/132 (↓)	↑ Fitm2, Rbfox1, Sgk3, and Foxo3	Apoptosis and atrophy	[113]

↑, activation; ↓, suppression; AKT, Akt kinase; ALK, anaplastic lymphoma kinase; AMPK, adenosine monophosphate-activated kinase; ARC, apoptosis repressor with caspase recruitment domain; ATF4, activating transcription factor 4; Bax, BCL2 associated X, apoptosis regulator; Bcl-2, B-cell leukemia/lymphoma 2; BIRC5, survivin, baculoviral inhibitor of apoptosis repeat-containing 5; BMPR1a, BMP type 1 receptors; BTG2, B-cell translocation gene 2; CHOP, C/EBP homologous protein; Drp-1, dynamin-related protein 1; eNOS, endothelial nitric oxide synthase; ER, endoplasmic reticulum; ErbB4, erb-b2 receptor tyrosine kinase 4; ERK, extracellular signal-regulated kinase; ETS1, E26 transformation-specific 1; Fitm2, transmembrane protein 2; FOXO, forkhead box class O; GATA-4, GATA binding protein 4; GRP78, 78-kDa glucose-regulated protein; GSDMD, Gasdermin D; HMGB1, high mobility group box 1; IL, interleukin; Keap1, Kelch-like ECH-associated protein 1; LDH, lactate dehydrogenase; LKB1, liver kinase B1; Mfn1, mitofusion-1; miR, microRNA; mTOR, mammalian target of rapamycin; mTORC1, mechanistic target of rapamycin complex 1; NF-κB, nuclear factor kappa B; NLRP3, NLR family pyrin domain-containing 3; Nrf2, nuclear factor erythroid 2-related factor 2; ORP8, oxysterol-binding protein-related protein 8; PDK1, 3-phosphoinositide-dependent protein kinase 1; PGC-1α, peroxisome proliferator-activated receptor gamma coactivator 1-alpha; PI3K, phosphoinositide 3-kinase; PPAR-γ, peroxisome proliferator-activated receptor gamma; PPIA, peptidylprolyl isomerase A; PTEN, phosphatase and tensin homolog; QKI, Quaking; Rbfox1, RNA-binding Fox protein 1; RhoH, Ras Homolog Family Member H; SERCA2a, sarco-endoplasmic reticulum Ca2+ ATPase; Sgk3, glucocorticoid-induced kinase 3; SIRT, silent information regulator 2 protein; TBP, TATA-Binding Protein; TfR1, transferrin receptor 1; VEGF-A, vascular endothelial growth factor-A; Wip1, wild-type p53-inducible protein 1; YES1, YES Proto-Oncogene 1; β1-AR, beta-1 adrenergic receptor.

## Abbreviations

AKT	Akt kinase
ALK	anaplastic lymphoma kinase
AMPK	adenosine monophosphate-activated kinase
AP-1	activating protein-1
ARC	apoptosis repressor with caspase recruitment domain
ASMCs	aortic smooth muscle cells
ATF4	activating transcription factor 4
AXIN2	axis inhibition protein 2
BACH1	BTB domain and CNC homolog 1
Bax	BCL2 associated X, apoptosis regulator
Bcl-2	B-cell leukemia/lymphoma 2
BIRC5	survivin, baculoviral inhibitor of apoptosis repeat-containing 5
BMP	bone morphogenetic protein
BMPR1a	BMP type 1 receptors
BTG2	B-cell translocation gene 2
CaSR	calcium-sensing receptor
CD	cluster designation
CHD9	Chromodomain Helicase DNA Binding Protein 9
CHOP	C/EBP homologous protein
C-kit	KIT pro-to-oncogene, receptor tyrosine kinase
COL1A1	collagen type I alpha 1 chain
COX-2	cyclooxygenase-2
CREG	Cellular Repressor of E1A-stimulated Genes
CUL2	Cullin-2
Cx43/Cx45	connexin 43/45
DOCK	dedicator of cytokinesis protein
DOX	doxorubicin
Drp-1	dynamin-related protein 1
ECs	endothelial cells
Elk	ETS-domain transcription factor
eNOS	endothelial nitric oxide synthase
ER	endoplasmic reticulum
ErbB4	erb-b2 receptor tyrosine kinase 4
ERK	extracellular signal-regulated kinase
Est1	SULT1E1, sulfotransferase family 1E member 1
ET-1	endothelin 1
ETS1	E26 transformation-specific 1
FGF	fibroblast growth factor
FGFR1	fibroblast growth factor receptor 1
Fitm2	transmembrane protein 2
FNDC5	fibronectin type III domain-containing protein 5
FOXO	forkhead box class O
GATA-4	GATA binding protein 4
GRP78	78-kDa glucose-regulated protein
GSDMD	Gasdermin D
HIF-1α	hypoxia-inducible factor 1-alpha
HMGB1	high-mobility group box 1
ICAM	Inter-Cellular Adhesion Molecule 1
IL	interleukin
Jag-1	jagged canonical Notch ligand 1
JNK1/2	c-jun-N-terminal kinase 1/2
Keap1	Kelch-like ECH-associated protein 1
KLF	Krüppel-like transcription factor
LATS2	large tumor suppressor kinase 2
LDH	lactate dehydrogenase
LKB1	liver kinase B1
LMOD1	leiomodin1
MAPK	mitogen-activated protein kinase
MEF2C	myocyte enhancer factor 2C
Mfn1	mitofusion-1
miR	microRNA
MMP2	matrix metallopeptidase 2
Mst1	macrophage stimulating 1
mTOR	mammalian target of rapamycin
mTORC1	mechanistic target of rapamycin complex 1
MYOCD	myocardin
NF-κB	nuclear factor kappa B
NLRP3	NLR family pyrin domain containing 3
Notch	Notch signal pathway
NR4A3	nuclear receptor 4A3
Nrf2	nuclear factor erythroid 2-related factor 2
ORP8	oxysterol-binding protein-related protein 8
p21/Cip1	cyclin-dependent kinase inhibitor 1A
p57/Kip2	cy-clin-dependent kinase inhibitor 1C
PAK4	p21 activated kinase 4
p-AKT	phosphorylated AKT
PASMCs	pulmonary artery smooth muscle cells
PCNA	proliferating cell nuclear antigen
PDGF-R	platelet-derived growth factor receptor
PDK1	3-phosphoinositide-dependent protein kinase 1
PGC-1α	peroxisome proliferator-activated receptor gamma coactivator 1-alpha
PI3K	phosphoinositide 3-kinases
PPAR-γ	peroxisome proliferator-activated receptor gamma
PPIA	peptidylprolyl isomerase A
PTEN	phosphatase and tensin homolog
PUMA	p53 upregulated modulator of apoptosis
PY-CARD	apoptosis-associated speck-like protein containing a CARD
QKI	Quaking homolog,
Rbfox1	RNA binding fox-1 homolog
RASA1	RAS P21 protein Activator 1
RhoH	Ras Homolog Family Member H
ROS	reactive oxygen species
RUNX2	Runt-related transcription factor 2
S1P	sphingosine-1-phosphate
SDC2	syndecan 2
SERCA2a	sarco-endoplasmic reticulum Ca2+ ATPase
Sgk3	glucocorticoid-induced kinase 3
SHR	spontaneously hypertensive rats
SIRT	silent information regulator 2 protein
SMCs	smooth muscle cell
SOCS2	suppressor of cytokine signaling 2
SphK1	sphingosine kinase 1
STAT	signal transducer and activator of transcription
TBP	TATA-binding protein
TfR1	transferrin receptor 1
TGF-β1	transforming growth factor beta 1
TNF-α	tumor necrosis factor α
TRB3	Tribbles-like protein 3
TSPAN2	tetraspanin 2
VCAM	vascular cell adhesion molecule
VEGFR	vascular endothelial growth factor receptor
VSMCs	vascular smooth muscle cells
Wip1	wild-type p53-inducible protein 1
YES1	YES Proto-Oncogene 1
ZO-1	zonula occludens-1
α-SMA	alpha-smooth muscle actin
β1-AR	beta-1 adrenergic receptor
